# A case-control study of rheumatoid arthritis identifies an associated single nucleotide polymorphism in the *NCF4 *gene, supporting a role for the NADPH-oxidase complex in autoimmunity

**DOI:** 10.1186/ar2299

**Published:** 2007-09-26

**Authors:** Lina M Olsson, Anna-Karin Lindqvist, Henrik Källberg, Leonid Padyukov, Harald Burkhardt, Lars Alfredsson, Lars Klareskog, Rikard Holmdahl

**Affiliations:** 1 Medical Inflammation Research, Lund University, BMC I11, 221 84, Lund, Sweden; 2 Cartela AB, Box 709, SE-220 07 Lund Sweden; 3 Institute for Environmental Medicine, Karolinska Institutet, Box 210, 171 77, Stockholm, Sweden; 4 Rheumatology Unit, Department of Medicine, Karolinska Institutet, 171 76, Stockholm, Sweden; 5 Division of rheumatology, Johann Wolfgang Goethe University, Theodor-Stern-Kai, 60596 Frankfurt am Main, Germany

## Abstract

Rheumatoid arthritis (RA) is a chronic inflammatory disease with a heritability of 60%. Genetic contributions to RA are made by multiple genes, but only a few gene associations have yet been confirmed. By studying animal models, reduced capacity of the NADPH-oxidase (NOX) complex, caused by a single nucleotide polymorphism (SNP) in one of its components (the *NCF1 *gene), has been found to increase severity of arthritis. To our knowledge, however, no studies investigating the potential role played by reduced reactive oxygen species production in human RA have yet been reported. In order to examine the role played by the NOX complex in RA, we investigated the association of 51 SNPs in five genes of the NOX complex (*CYBB*, *CYBA*, *NCF4*, *NCF2*, and *RAC2*) in a Swedish case-control cohort consisting of 1,842 RA cases and 1,038 control individuals. Several SNPs were found to be mildly associated in men in *NCF4 *(rs729749, *P *= 0.001), *NCF2 *(rs789181, *P *= 0.02) and *RAC2 *(rs1476002, *P *= 0.05). No associations were detected in *CYBA *or *CYBB*. By stratifying for autoantibody status, we identified a strong association for rs729749 (in *NCF4*) in autoantibody negative disease, with the strongest association detected in rheumatoid factor negative men (CT genotype versus CC genotype: odds ratio 0.34, 95% confidence interval 0.2 to 0.6; *P *= 0.0001). To our knowledge, this is the first genetic association identified between RA and the NOX complex, and it supports previous findings from animal models of the importance of reactive oxygen species production capacity to the development of arthritis.

## Introduction

Rheumatoid arthritis (RA) is a chronic inflammatory disease that leads to erosion and deformation of the joints. The prevalence in the general population is 0.5% to 1%, and women are at two to three times greater risk for developing the disease. Twin studies show a concordance rate of 12% to 15% in monozygotic twins and 4% in dizygotic twins, and the genetic heritability is estimated at 60% [1]. Despite much effort to identify arthritis causing genes, only few genetic loci have been confirmed to be associated with RA, among which are the human leucocyte antigen (*HLA*) locus and the protein tyrosine phosphatase non-receptor 22 gene (*PTPN22*) [2-4].

RA is a heterogeneous disease with symptoms and disease progression that vary between patients. Classification of typical RA requires fulfilment of four out of seven criteria established by the American College of Rheumatology [5]. The heterogeneity of the symptoms in RA probably mirrors the underlying genetic contribution; hence, the various combinations of symptoms observed in patients are probably caused by different combinations of risk alleles.

In the search for markers that can predict development of the disease before its onset, several autoantibodies, including rheumatoid factors (RFs) and antibodies against cyclic citrullinated peptides (anti-CCP antibodies), have been detected [6-8]. RFs, autoantibodies that recognize the Fc part of immunoglobulins [9], are present in 60% to 70% of RA patients and have been found to be associated with more severe clinical manifestations [10,11]. Anti-CCP antibodies are present in 50% to 60% of RA patients and have also been shown to predict more severe disease [10,12-14]. Interestingly, recent data suggest that the *HLA-DRB1 *locus, which has been shown to be associated with RA, is only associated with the presence of anti-CCP antibodies, and this association is independent of both RA development and the presence of RFs [15]. Together with recent data describing the interaction between environmental factors and genetic predisposition [16,17], these findings support the hypothesis that several independent disease mechanisms may lead to development of RA. They also emphasize the need to use relevant subgroups of RA patients in genetic association studies.

Various approaches have been used to identify genes that contribute to common diseases such as RA [18,19]. Because of increasing knowledge of gene functions and immunological pathways, a candidate gene approach can be more efficient in terms of both cost and time than traditional linkage analysis or genome-wide approaches. The selection of genes in a candidate gene study can be based on previous knowledge of gene functions as well as on disease or immunological mechanisms. It can also be based on findings in animal models.

The *Pia4 *locus in rats has been shown to reduce the severity of pristane-induced arthritis (an arthritis model) [20]. A single nucleotide polymorphism (SNP) in the *Ncf1 *gene was found to be responsible for the protective effect [21]. *Ncf1 *encodes the protein p47^phox^, which is part of the NADPH-oxidase (NOX) complex that produces reactive oxygen species (ROS) in response to infectious stimuli. Rats carrying the risk allele have reduced capacity to produce ROS, which is linked to increased risk for development of arthritis [21,22]. Apart from *NCF1*, the NOX complex is composed of five other proteins encoded by the genes *NCF2*, *NCF4*, *CYBB*, *CYBA *and *RAC2 *[23,24]. In phagocytes massive ROS production, called the oxidative burst, takes place in the phagosome or endosome (intracellular) or in the plasma membrane (extracellular) after ingestion of invading pathogens or after stimulation of innate immune receptors [25-27]. However, the ability to generate ROS extends to cells other than classical phagocytes, such as dendritic cells, suggesting that the NOX complex has additional functions in the immune system [28]. Interestingly, recent findings from our group show that the redox balance of T-cell membranes has an important effect on the activation and proliferation of T cells [29].

Results obtain in animal models make *NCF1 *and the other genes of the NOX complex candidate genes for human RA. However, transferring animal data to the human setting is not straightforward in this case. In contrast to rats, the genomic organization of *NCF1 *in humans is very complex, which makes SNP-based association analysis difficult [30,31]. However, because of the complex interplay between the proteins of the NOX complex, it is likely that genetic changes in any of the genes could have the same effect on ROS production, as is seen for *Ncf1 *in rats.

We used a candidate gene approach to study the association with RA of 51 SNPs in five genes included in the NOX complex. We found a SNP, located in intron 4 in *NCF4*, to be associated with RA in a Swedish case-control cohort. This supports the hypotheses that the NOX complex is involved in the development of RA and that ROS could be an important regulator of the immune system.

## Materials and methods

### Selection of single nucleotide polymorphism

The HapMap genome browser [32] was used as the primary source for selection of SNPs. Primarily, HapMap-validated SNPs were selected. However, in order to obtain evenly dispersed SNPs across the genes, SNPs were also selected from dbSNP [33]. The selection was based on several criteria; the minor allele frequency should be more than 5% in the Centre d'Etude du Polymorphisme Humain (CEPH) or other European cohort, the SNP should be located in a nonrepetitive sequence, and the validation status at dbSNP should be at least two-hit. We also consulted the Linkage disequilibrium (LD) maps of the HapMap CEPH samples in order to disperse evenly the SNPs with respect to the LD structure. Sixty-seven SNPs were selected (see Additional file 1): six in *CYBB*, eight in *CYBA*, 22 in *NCF2*, 21 in *NCF4 *and 10 in *RAC2*. The number of SNPs selected in each gene reflects both the size of the gene and the availability of validated SNPs.

### Samples

Samples from the Epidemiological Investigation of Rheumatoid Arthritis (EIRA), a population based case-control study, were analyzed. The study base comprised the population, aged 18 to 70 years, in a geographically defined area in the middle and southern parts of Sweden during the period from May 1996 to 2005. A case was defined as a person in the study base who, during the study period and for the first time, received a diagnosis of RA based on the American College of Rheumatology criteria of 1987 [5]. All cases were examined and diagnosed by a rheumatologist at a participating centre. All public rheumatology units in the study area and almost all of the (very few) private units participated in the study. The present study includes data from 1,842 RA patients and 1,038 controls, randomly selected from the study base and matched on age, sex and residential area. Of the cases and controls, 71% and 72%, respectively, are female; the mean age was 51 ± 13 years in cases and 54 ± 12 in controls. Information about RF and anti-CCP antibody status was available for 1,315 of the patient samples; 63% of these were RF positive and 61% were anti-CCP positive.

### Genotyping

Assay design, validation and genotyping were performed by Kbiosciences (London, UK) using a fluorescence resonance energy transfer based competitive allele-specific polymerase chain reaction system (KASPar).

Of the selected 67 SNPs, 51 were successfully turned into assays. Six SNPs failed to make good assays (rs1049255 [*CYBA*], rs699244, rs789180, rs4652813 and rs6667363 [*NCF2*], and rs2075938 [*NCF4*]), whereas 10 were monomorphic in the panel of 40 Caucasians used for validation or in the EIRA cohort (rs1804006 [*CYBA*], rs789183, rs13306581 and rs13306575 [*NCF2*], and rs13057803, rs1003501, rs12158689, rs9610595, rs2072706 and rs2072711 [*NCF4*]; Additional file 1). Fourteen of the SNPs were genotyped in all available samples (1,842 cases and 1,038 controls), whereas the other 37 SNPs were genotyped in a subset of the samples comprising 1,069 patients and 634 controls (Additional file 1).

### Hardy-Weinberg analysis

The Haploview software calculates *P *values for deviations from Hardy-Weinberg equilibrium (HWE) for each marker on the complete uploaded dataset [34]. A significance threshold of *P *≤ 0.001 was used. In order to obtain HWE *P *values for the case and control groups separately, each dataset was uploaded separately.

### Single marker analysis

Contingency tables were created for each SNP using the JMP 5.0 software (SAS Institute, Cary, NC, USA), and *P *values for association with RA were calculated using χ^2 ^tests for genotype frequencies. The sample set was stratified for sex in the initial analysis, and for sex and RF or anti-CCP status in the stratified analysis. Because *CYBB *is located on the X chromosome, allele frequencies were used to estimate association in the male samples.

To obtain a corrected α level in the stratified analysis, the Bonferroni correction method was applied, as implemented on the simple interactive statistical analysis website [35].

We also performed logistic regression analysis for the rs729749 SNP, in order to adjust for age, sex and living area. Logistic regression analysis was conducted for a subset of the material, in which all information regarding genetic factors, antibodies and matching variables was available. This same sample set was used for the frequency analysis of rs729749. We used the SAS software for Windows (version 9.1; SAS Institute, Cary, NC, USA) to perform logistic regression analysis.

### Haplotype analysis

Haplotype blocks were calculated using Haploview [34]. SNPs that departed from HWE in both cases and controls were excluded from the analysis. The haplotype predictions were based on the CI method proposed by Gabriel and coworkers [36]. However, the other available methods yielded the same result. Haplotypes were assessed using the WHAP software [37]. Sub-haplotypes were investigated for associations based on the block structure predicted in Haploview. Because the haplotype block analysis is based on all genotyped SNPs, we used only the genotype information from the subset of the EIRA cohort comprising 1,069 cases and 634 controls. The permuted *P *values for the haplotypes are based on 5,000 permutations.

### Genome analysis

The region surrounding rs729749 was investigated for the presence of regulatory elements, transcription factor binding sites and conserved regions using the University of California, Santa Cruz (UCSC) Genome Browser [38]. The ESPERR Regulatory potential (seven species), Conservation, and TFBS Conservation options were used.

*P *values and odds ratios (OR) with 95% CIs for the genetic models were calculated using the GraphPad Prism software (GraphPad Software, San Diego, CA, USA).

## Results

### Fifty-one single nucleotide polymorphisms were successfully genotyped in a Swedish rheumatoid arthritis cohort

To investigate the genes in the NOX complex for association with RA, 67 SNPs evenly dispersed in the five candidate genes (Table 1) were selected from the HapMap genome browser and dbSNP (National Center for Biotechnology Information; Additional file 1). Fifty-one out of the 67 SNPs were successfully assayed and genotyped in the Swedish EIRA cohort [39,40], with a 98% call rate (Additional file 1). Deviations from HWE were estimated for each SNP in all samples combined and in cases and controls separately. The SNP rs3788524 (*NCF4*) significantly deviated from HWE (*P *< 0.001) in both the case and control groups, indicating a possible genotype failure.

**Table 1:** Gene positions

Gene	Chromosome	Position	Exons (*n*)
*NCF2*	1	181,791,321 to 181,826,339	15
*NCF4*	22	35,586,991 to 35,604,004	10
*CYBB*	X	37,524,264 to 37,557,658	13
*CYBA*	16	87,237,199 to 87,244,958	6
*RAC2*	22	35,951,258 to 35,970,251	7

Positions according to National Center for Biotechnology Information build 36.1 from the University of California, Santa Cruz genome browser.

### Genotype analysis indicates male-specific associations with rheumatoid arthritis in *NCF2*, *NCF4 *and *RAC2*

To evaluate the SNPs for association with RA, contingency tables were created for genotype frequencies and *P *values were calculated using χ^2 ^tests. Because RA is more frequent in women than in men, the disease mechanisms might be sex dependent. The samples were therefore analyzed either all together or stratified by sex. The initial cut-off *P *value for significance was set at 0.05 to reduce the risk for missing subgroup-specific associations, which would be weak in the complete sample set. Three SNPs fulfilled this criterion for association with RA, all in men; rs789181 in *NCF2 *(*P *= 0.03), rs729749 in *NCF4 *(*P *= 0.001) and rs1476002 in *RAC2 *(*P *= 0.05; Table 2).

**Table 2:** Associated SNPs in the initial genotype analysis

SNP id	Case/control	Genotype			Sex	*P*
rs729749	Case	CC 0.69 (256)	CT 0.26 (95)	TT 0.05 (18)	Male	0.001
	Control	CC 0.57 (141)	CT 0.40 (98)	TT 0.04 (9)		
rs789181	Case	AA 0.81 (244)	AG 0.15 (46)	GG 0.03 (10)	Male	0.03
	Control	AA 0.78 (146)	AG 0.22 (41)	GG 0.005 (1)		
rs1476002	Case	CC 0.77 (395)	CT 0.22 (115)	TT 0.01 (6)	Male	0.05
	Control	CC 0.76 (229)	CT 0.20 (61)	TT 0.04 (11)		

SNP, single nucleotide polymorphism. Values indicate the genotype frequencies and number (in brackets).

### Stratification based on autoantibody status reveals a highly significant association of rs729749 with rheumatoid factor negative rheumatoid arthritis in men

Because RA is a heterogeneous disease, it is likely that the disease contributing genes differ in different subgroups of patients. Presence or absence of autoantibodies such as RF and anti-CCP antibodies might reflect different underlying disease mechanisms. In order to determine whether the detected associations are specific for a subclass of RA, we stratified the male cases by RF or anti-CCP antibody status and performed an analysis on genotype frequencies for the associated SNPs.

We found the SNPs rs789181 (*NCF2*) and rs1476002 (*RAC2*) to be significantly associated in RF-positive men (*P *= 0.03 and 0.02, respectively). The SNP rs1476002 was also significantly associated in anti-CCP antibody positive men (*P *= 0.05). However, the *P *values are only slightly improved compared with the initial analysis, suggesting that the associations are not specific for any of these subgroups. On the other hand, the association for rs729749 in *NCF4 *is highly significant in RF-negative men (*P *= 0.0002), whereas no association was detected in RF-positive men (*P *= 0.0713; Table 3). Furthermore, a comparison of the frequencies in RF-negative against RF-positive men also yielded a significant association with RF-negative disease (*P *= 0.01). We also detected a weaker association in anti-CCP antibody negative men (*P *= 0.003; Table 3), although the anti-CCP antibody negative versus anti-CCP antibody positive comparison is not significant (*P *= 0.08). Logistic regression analysis of the CT or TT genotype against the CC genotype, adjusted for age and residential area, also indicated a sex and subgroup specific effect for rs729749 (Table 4). With regard to RF-negative RA among men, the OR for CT against CC is 0.34 (95% CI = 0.20 to 0.60; *P *= 0.0001). Here we also see a strong association in anti-CCP antibody negative men (OR = 0.4, 95% CI = 0.2 to 0.7; *P *= 0.0004).

**Table 3:** Stratified analysis of rs729749 in men

Antibody status	Case/control	Genotypes			*P*
			
		CC	CT	TT	
RF positive	Case	0.68 (169)	0.29 (71)	0.03 (7)	0.0713
	Control	0.58 (170)	0.37 (108)	0.04 (11)	
RF negative	Case	0.71 (87)	0.20 (24)	0.09 (11)	0.0002
	Control	0.57 (141)	0.40 (98)	0.04 (9)	
Anti-CCP positive	Case	0.69 (159)	0.28 (63)	0.03 (7)	0.0398
	Control	0.59 (167)	0.38 (107)	0.04 (11)	
Anti-CCP negative	Case	0.70 (97)	0.22 (31)	0.08 (11)	0.0032
	Control	0.59 (167)	0.38 (107)	0.04 (11)	

CCP, cyclic citrullinated peptide; RF, rheumatoid factor. Values indicate the genotype frequencies and number (in brackets).

**Table 4:** Logistic regression analysis of rs729749 in cases stratified for autoantibody status

Status	Subgroup	Genotype CT versus CC			Genotype TT versus CC		
		
		OR	95% CI	*P*	OR	95% CI	*P*
All	All	0.80	0.66 to 0.97	0.02	1.04	0.67 to 1.62	0.86
	Women	0.94	0.75 to 1.17	0.56	1.01	0.60 to 1.71	0.97
	Men	0.50	0.35 to 0.73	0.0002	1.20	0.52 to 2.81	0.67
RF positive	All	0.85	0.69 to 1.04	0.12	0.92	0.56 to 1.53	0.76
	Women	0.96	0.75 to 1.23	0.74	1.03	0.58 to 1.84	0.92
	Men	0.59	0.39 to 0.88	0.009	0.74	0.26 to 2.12	0.58
RF negative	All	0.70	0.54 to 0.90	0.006	1.24	0.71 to 2.14	0.45
	Women	0.88	0.65 to 1.18	0.39	0.94	0.47 to 1.90	0.87
	Men	0.34	0.20 to 0.60	0.0001	2.03	0.88 to 5.29	0.15
Anti-CCP positive	All	0.86	0.70 to 1.07	0.17	1.01	0.61 to 1.71	0.98
	Women	1.02	0.80 to 1.31	0.88	1.12	0.63 to 2.00	0.70
	Men	0.54	0.36 to 0.83	0.003	0.79	0.28 to 2.24	0.65
Anti-CCP negative	All	0.69	0.54 to 0.89	0.004	1.07	0.62 to 1.84	0.82
	Women	0.82	0.61 to 1.10	0.19	0.86	0.43 to 1.73	0.67
	Men	0.4	0.2 to 0.7	0.0004	1.8	0.7 to 4.6	0.26

CCP, cyclic citrullinated peptide; CI, confidence interval; OR, odds ratio; RF, rheumatoid factor.

To determine the validity of the *P *value, we estimated a corrected α value using the Bonferroni correction method. The corrected α level for the number of tests performed (159) was estimated at 0.0003. Hence, only the association detected for rs729749 in RF-negative males remained significant using the corrected α level.

### Haplotype analysis reveals an associated haplotype in *NCF4 *caused by the rs729749 single nucleotide polymorphism

**Figure 1:**
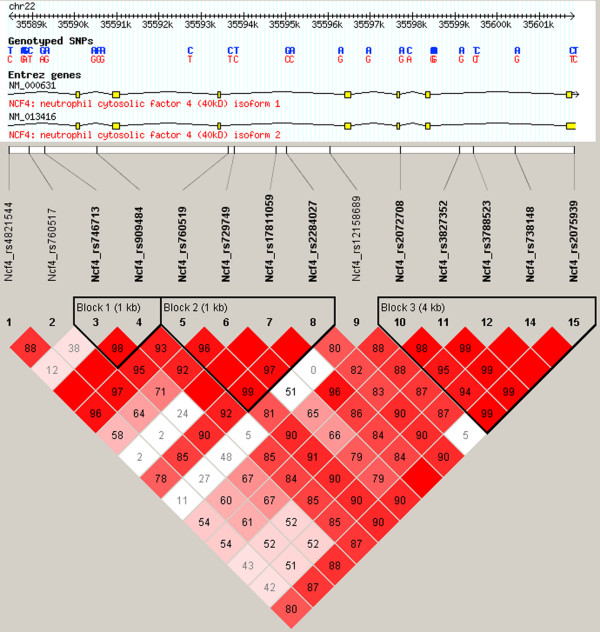
Haplotype blocks in *NCF4*. Three haplotype blocks were identified in *NCF4*. Block 2 contains the associated single nucleotide polymorphism (SNP) rs729749. Colour scheme of the linakge disequilibrium LD map is based on the standard D'/LOD option in the Haploview software. The LD blocks are calculated based on the CI method.

We wished to estimate haplotypes and haplotype blocks to determine whether rs729749 is included in a conserved and possibly associated haplotype. The Haploview software was used to determine the haplotype block structures of the genes. The analysis showed that the *NCF4 *gene is divided into three blocks (Figure 1). One block of 1 kilobase contains rs729749 as well as three other genotyped SNPs: rs760519, rs2284027 and rs17811059. To test the association of the haplotype blocks, we used the WHAP software. The block containing rs729749 is borderline significant in the nonstratified analysis (*P *= 0.055), but excluding rs2284027 and rs17811059 yields a significant association (empirical *P *= 0.04 in all and 0.03 in men; Table 5). In order to determine how important the impact of the rs729749 SNP is for the identified associated haplotype, we conducted a conditional analysis in which the association of the haplotype was estimated while controlling for the haplotype background of rs760519 and rs2284027. This test gave an empirical *P *value of 0.01, which is lower than for the associated haplotype, indicating that the rs729749 SNP itself or a SNP in strong linkage disequilibrium is causing the haplotype association.

**Table 5:** Haplotype analysis

Haplotype^a^	Frequency		Subgroup	Empirical *P*^b^
			
	Cases	Controls		
TC	0.82	0.80	-	0.038
CT	0.15	0.16		
TT	0.04	0.05		
TC	0.81	0.77	men	0.029
CT	0.15	0.17		
TT	0.03	0.06		

^a^Haplotype of rs760519 and rs729749. ^b^Based on 5,000 permutations.

### Homozygosity could explain the genetic risk associated with rs729749

The rs726749 SNP is noncoding and located in the beginning of intron 4 in *NCF4*. Analysis of the region using the UCSC genome browser (March 2006 assembly) [38] revealed that it is not located at any known transcription factor binding site or regulatory element; however, there is a conserved regulatory region (as predicted by the ESPERR regulatory potential option) approximately 300 base pairs downstream of rs729749. Nonetheless, the genetic effect of this SNP is not obvious.

By looking at the genotype frequencies in RF-negative males for rs729749, it appears as though both homozygous genotypes are more common in the RA patients (Table 3). This led us to perform an analysis to determine which genetic model best fits the data. Not surprisingly, the homozygous model (CC + TT versus CT) gave the best fit in the subgroup of RF-negative men (OR = 2.67, 95% CI = 1.60 to 4.46; *P *= 0.0002; Table 6). However, because the allele frequency of the T-allele is quite low and few individuals are homozygous for this allele, there is a degree of uncertainty in this prediction. The CC genotype is clearly more common in the patients and homozygosity at this locus also gives a significant genetic model (Table 6).

**Table 6:** Genetic models of rs729749 in RF negative males

Model	Frequency of risk variant		OR	95% CI	*P*
				
	Cases	Controls			
CC + TT versus CT	0.80	0.60	2.67	1.60 to 4.46	0.0002
CC versus CT	0.71	0.57	2.52	1.50 to 4.24	0.0004
TT versus CT	0.09	0.04	4.99	1.86 to 13.4	0.0016
CC versus CT + TT	0.71	0.57	1.89	1.18 to 3.01	0.0088
TT versus CC + CT	0.09	0.04	2.63	1.06 to 6.53	0.0476

CI, confidence interval; OR, odds ratio; RF, rheumatoid factor.

## Discussion

Here we report an association of the SNP rs729749, located in the gene *NCF4*, with RA in Swedish RF/anti-CCP antibody negative male patients. *NCF4 *is part of the NOX complex, and our results support recent findings that ROS play a role in the development of RA. However, because this is the first report of an association between a gene in the NOX complex and RA, genetic replication as well as functional studies will be required before it is possible to determine conclusively whether *NCF4 *is an RA susceptibility gene. Furthermore, the results from this study do not exclude the possibility that the other genes in the NOX complex could affect RA susceptibility. There could, for example, be epistatic effects between the different genes in the complex.

There is a lack of consensus in the field about statistical significance levels in association studies. In this study we initially used an α level of 0.05 to reduce the risk for overlooking associations that are specific for a subgroup of RA patients. There are strong medical and biological arguments in favour of subgrouping RA patients, and an analysis of nonstratified samples might conceal a true association. In the initial analysis of the genotype frequencies we found indications of association with three SNPs, and stratification of the samples indicates that the association for rs729749 is male specific and predominates in RF-negative disease. We also found a weaker association in anti-CCP antibody negative disease, but at this stage it is not possible to say whether this is due to the fact that the presence of RFs and anti-CCP antibodies is dependent [41] or whether the anti-CCP antibodies independently influence genetic risk.

The detected association of rs729749 is strongest in RF-negative males, and therefore it is possible that this particular SNP modulates the affected disease pathway so that it mainly influences the clinical disease in RF-negative RA, specifically in men. However, it is not impossible that this pathway play a role also in other subtypes of RA. The results from this study strengthen the view that different combinations of genes are involved in the disease progression in different RA subclasses defined, for instance, by sex or autoantibody status. The most striking aspect of the identified association of the rs729749 SNP is the clear sex specificity, indicating that the disease pathway affected by the rs729749 SNP is specific to men. The fact that RA is three times more common in women than in men suggests that men and women respond differently to factors that trigger the onset of RA and that some disease pathways could be sex specific. The RF-negative specificity of the association is not as striking as the sex specificity, but it might still point toward which RA subgroup is affected by the rs729749 SNP. Because both RFs and anti-CCP antibodies can be detected before the appearance of any disease symptoms [7,42], they might reflect or be part of initial disease mechanisms that are only present in anti-CCP antibody or RF positive patients. Furthermore, it has repeatedly been shown that patients who are negative for these autoantibodies have a less severe disease outcome than do anti-CCP antibody or RF positive RA patients [10-12]. This further supports the view that autoantibody negative RA is another variant of RA. Also, the few genes confirmed to be associated with RA have been shown mainly to affect subclasses of RA.

The well established association of the *HLA-DRB1 *alleles, which encode the so-called shared epitope (SE), was recently suggested to be restricted to anti-CCP antibody but not RF positive RA [15,16,41]. However, it has also been shown that SE alleles are associated with higher titres of autoantibodies against type II collagen [43]. Interestingly, the *HLA-DR3 *locus, which does not encode the SE, appears to be associated only with anti-CCP negative RA [41,44]. The recently discovered *PTPN22 *risk allele has been shown to be associated with both RF and anti-CCP antibody positive RA [43,45,46], but there are conflicting data regarding the autoantibody restriction of this association [47]. Furthermore, the reported association of the PD-1.2A allele of the *PDCD1 *gene is restricted to RF negative as well as SE negative RA [48]. Hence, even though little is known about the precise disease mechanisms in RA, it is obvious that the symptoms can be caused by several different distinct disease pathways.

Because of the lack of certain autoantibodies, it is tempting to speculate that RF or anti-CCP antibody negative RA patients have a lesser B-cell component, and reports indicating that B-cell depleting therapy is a less effective treatment for RF-negative RA supports such a view [49,50]. Several recent clinical studies, however, have yielded conflicting results on B-cell depletion efficiency [51], and more extensive studies are required before any conclusions can be drawn about the B-cell dependence in RF and anti-CCP antibody negative RA. In addition, it is fair to assume that the autoantibody negative forms of RA, in themselves, represent a heterogeneous collection of pathways related, for example, to osteoarthritis, arthropathies, or lupus.

The rodent arthritis models are heterogeneous as well but they may relate to more defined arthritis conditions than the human disease. Interestingly, the pristane-induced arthritis model used to identify the polymorphism in *Ncf1 *in rats is B-cell independent. Studies have shown that transfer of T-cells from genetically susceptible and immunized rats into genetically resistant and un-immunized rats is enough to induce arthritis in the resistant rat [52].

Both the *HLA-DRB1 *locus and *PTPN22 *are believed to affect T-cell activation. *HLA-DRB1 *is expressed by antigen-presenting cells and restricts the presentation of antigens to T cells. *PTPN22 *on the other hand has an intracellular effect, and the amino acid shift to the disease-associated tryptophan impairs the function of the Lyp protein, encoded by *PTPN22*, rendering it a less potent negative regulator of T-cell activation [53]. Studies in animal models as well as recent findings suggest that the NOX complex could also be involved in T-cell activation [29,54]. ROS production has been found in antigen-presenting cells, and it has also been shown that T-cell receptor signalling is affected by oxidation of the T-cell membrane as well as by intracellular levels of ROS [55,56]. Interestingly, hydrogen peroxide generated from ^•^O_2_^- ^produced by the NOX complex can readily cross the cell membrane and inactivate protein tyrosine phosphatases, including that encoded by *PTPN22*, through oxidation of a specific cysteine residue [54]. ROS production of antigen-presenting cells, such as dendritic cells, has been found to be crucial for the activation of T-cells [28] and could therefore determine the immune response to an antigen. Furthermore, recent work from our group highlights the importance of the redox balance on the surface of T cells for activation. T-cells originating from the *Ncf1 *mutated rat DA have an increased number of reduced thiol groups on the cell surface, which increases proliferation and activation [29]. Gelderman and coworkers [29] showed that T-cells originating from the nonmutated E3 rats could be made arthritogenic by increasing the number of reduced thiol groups on the cell surface. Because NOX production could not be detected in T-cells, it was concluded that the redox balance of T-cell surfaces is determined by other cells, such as macrophages or dendritic cells.

In the rat a clear difference in the capacity to produce oxidative burst is the result of a single SNP in *Ncf1*, resulting in the shift from threonine to the disease-promoting methionine at position 153 in the p47^phox ^protein [21]. The consequences of the amino acid shift have not yet been identified, although position 153 does not coincide with any critical binding sites. It is most likely that the shift affects the three-dimensional structure of the protein, impairing the binding capabilities to the other proteins in the complex. The activation of the NOX complex is initiated through phosphorylation of the three cytoplasmic proteins p47^phox ^(*NCF1*), p67^phox ^(*NCF2*) and p40^phox ^(*NCF4*) [23,26,57]. The phosphorylations lead to conformational changes of p47^phox^, and subsequent translocation of the three proteins to the membrane, where it interacts with and activates the membrane bound complex flavocytochrome b_558_, which is composed of gp91^phox ^(*CYBB*) and p22^phox ^(*CYBA*) [24,58]. *RAC2 *also translocates to the membrane after dissociation from Rho-guanine nucleotide dissociation inhibitor and interacts with flavocytochrome b_558 _[23,59]. Studies show that p67^phox ^and Rac2 are critical for the function of the complex [25,60], whereas p40^phox ^and p47^phox ^function as adaptor proteins and are responsible for the assembly of the complex [61]. The precise role of p40^phox ^is still debated, and there is evidence of both positive and negative regulation of the NOX complex [61-63]. Recent studies have suggested that p40^phox^, as well as p47^phox^, functions by 'carrying' p67^phox ^to the membrane through the interaction with phospholipids [62,64]. Studies of mutations causing human chronic granulomatous disease show that mutations in *NCF1*, *CYBB*, *CYBA*, or *NCF2 *lead to a complete lack of function of the NOX complex [65,66]. However, SNPs in less critical regions, like the 153 SNP in *Ncf1 *found in rat, apparently reduce but do not completely abolish ROS production, which leads to an increased susceptibility to arthritis [21]. These facts indicate that the SNPs of interest for examination in an RA association study of the NOX complex will probably be located at noncritical positions and will not completely abolish but only slightly modify binding to the other proteins.

The rs729749 SNP is located in the middle part of intron 4 in *NCF4*. By studying the genome maps using the UCSC genome browser, we could not find any evidence of it affecting a transcription factor binding site or a known regulatory element. However, there is a conserved regulatory region predicted approximately 300 base pairs downstream of rs729749. The haplotype analysis indicates that rs729749, or a SNP in very high LD, is responsible for the association detected for the haplotype. Also, because the predicted regulatory region is very close to rs729749, it could very well contain the causative SNP.

The genetic analysis of the association shows that the homozygous model gives the best significance, indicating that this could be the genetic effect. However, the low number of samples in the subgroup of RF-negative males together with the low frequency of the T allele make this assumption somewhat uncertain. Furthermore, it is not obvious what the functional consequences of a homozygous effect would be. We therefore chose to present all models tested in order to provide the full picture.

In the logistic regression analysis, weak associations were also detected for RF and anti-CCP antibody positive disease. One possible explanation for these findings is that some patients could have been 'misdiagnosed' regarding autoantibody status. The diagnosis is made by measuring the autoantibody titres, and certain threshold are used to determine positive versus negative status. However, sometimes autoantibody status changes as the disease progresses, and therefore there is a degree of uncertainty regarding autoantibody status.

A method or a strategy to apply multiple testing corrections accurately in a case-control candidate gene association study has not yet been established [67,68]. The conventional multiple correction methods, such as Bonferroni, are considered by some to be too stringent for large-scale studies [69,70]. The Bonferroni correction method is used to correct for the number of independent tests performed. However, in an association analysis neither the SNPs tested nor the different stratified analyses are truly independent. The allele frequencies of the SNPs are affected by the LD of the region, and the stratified analyses are based on the same SNPs and samples as the initial analysis and can therefore not be considered to be independent from each other. Nonetheless, in order to obtain an indication of the validity of the *P *values obtained in these analyses, we used the Bonferroni correction method to estimate an α level correcting for the number of tests (159) performed in the frequency analysis. The 159 tests reflect the sex-stratified analysis of all 51 SNPs (153) plus the stratified analysis of RF and anti-CCP antibody status performed for rs729749, rs789181 and rs1476002 in the male subset (6). Correcting for 159 tests gives a new α level of 0.0003, which means that only the association of rs729749 in RF-negative males passed the significance threshold using this stringent method.

The EIRA study included mainly individuals born in Sweden. Taking those born outside Sweden into consideration also, 97% were of Caucasian origin. In order to minimize potential bias resulting from population stratification, we performed logistic regression analyses in which ORs were adjusted for age and residential area.

## Conclusion

We found evidence of an RA-associated SNP in the *NCF4 *gene of the NOX complex. The association is specific for male patients, with the strongest association found in RF-negative RA. This finding supports the notion of RA being a heterogeneous disease caused by a variety of disease pathways that are regulated by a variety of contributing risk genes. The detected association with a component of the NOX complex also strengthens previous evidence obtained in animal models that suggests that the NOX complex and ROS have a major impact on inflammation and autoimmunity. It is hoped that this finding will help to elucidate the complex genetics that underlie RA and autoimmunity.

## Abbreviations

CCP = cyclic citrullinated peptide; CEPH = Centre d'Etude du Polymorphisme Humain; CI = confidence interval; EIRA = Epidemiological Investigation of Rheumatoid Arthritis; HWE = Hardy-Weinberg equilibrium; NOX = NADPH oxidase; OR = odds ratio; RA = rheumatoid arthritis; RF = rheumatoid factor; ROS = reactive oxygen species; SE = shared epitope; SNP = single nucleotide polymorphism.

## Competing interests

The authors declare that they have no competing interests.

## Authors' contributions

LMO has been the responsible investigator for the project and has carried out SNP selection, genotype preparation, statistical analyses and drafted the manuscript. A-KL participated in the design of the study and the statistical analyses. HK and LA performed the logistic regression analysis. LP and LK were responsible for the distribution of the EIRA cohort. HB has contributed with intellectual property to the study. RH conceived of the study, participated in its design and helped to draft the manuscript.

## Supplementary Material

Additional file 1 An Word file containing a table that shows a table of all SNPs evaluated for association with RA in this study. Click here for file
