# Supplementary material for: A case-control study of rheumatoid arthritis identifies an associated single nucleotide polymorphism in the NCF4 gene, supporting a role for the NADPH-oxidase complex in autoimmunity
Source: Arthritis Res Ther. 2007 Sep 26;9(5):R98. doi: 10.1186/ar2299 (PMC2212587; doi:10.1186/ar2299)
Supplement: Additional file 1 — An Word file containing a table that shows a table of all SNPs evaluated for association with RA in this study. [file ar2299-S1.doc]

### Supplementary table 1

^1^Minor allele first. ^2^Homozygous for the minor allele, heterozygous, homozygous for the major allele. ^3^Genotype frequencies from females, allele frequencies from males. ^*^Genotyped in the complete sample set. f = failed assay. mm = monomorphic. HWD = Hardy-Weinberg disequilibrium.
